# Preoperative of balloon pulmonary angioplasty in chronic thromboembolic pulmonary hypertension using virtual balloon expansion: a pilot study

**DOI:** 10.3389/fbioe.2026.1741437

**Published:** 2026-04-13

**Authors:** Qianwen Liu, Yonglei Zhao, Zeping Jiang, Hao Lu, Yuanming Luo, Renbiao Chen

**Affiliations:** 1 Department of Technology, Boea Wisdom (Hangzhou) Network Technology Co., Ltd., Hangzhou, China; 2 Department of Radiology, Sir Run Run Shaw Hospital (SRRSH), Zhejiang University School of Medicine, Hangzhou, China; 3 Department of Mechanical Engineering, The University of Iowa, Iowa City, IA, United States

**Keywords:** balloon pulmonary angioplasty, chronic thromboembolic pulmonary hypertension, computational fluid dynamics, hemodynamic, virtual surgery

## Abstract

**Objectives:**

Chronic thromboembolic pulmonary hypertension (CTEPH) demands precise balloon pulmonary angioplasty (BPA) planning to address unpredictable hemodynamic outcomes. This pilot study explores the potential of virtual simulation to inform this process.

**Methods:**

We developed two virtual BPA (vBPA) approaches for patient-specific computational fluid dynamics (CFD) models derived from preoperative computed tomography pulmonary angiography (CTPA) scans of three CTEPH cases featuring right-lung lesion dominant: vBPA1 for morphology-restoring dilation and vBPA2 for rigid homogeneous dilation. We simulated interventions on 28 vascular regions, generating 336 hemodynamic comparisons quantified via Euclidean distance and a composite score integrating multiple spatial metrics. The Euclidean distance value or composite score represents deviation from virtual POST.

**Results:**

Our analysis suggested a complex relationship between pulmonary vascular resistance (PVR) and vortex dynamics. The vBPA simulations indicated a temporal decoupling in hemodynamic response, where initial PVR improvement could coincide with an intensification of high-helicity vortices, potentially associated with subsequent hemodynamic rebound as observed in one patient. Preliminary trends hinted at differential performance of the vBPA methods across lesion types. vBPA2 (rigid dilation) showed relatively lower composite scores for web lesions (composite scores: 0.176–0.220 vs. vBPA1: 0.239–0.262), while vBPA1 (morphology-restoring) produced a relatively lower score for the ring-like stenosis (composite score: 0.168 vs. vBPA2: 0.190).

**Conclusion:**

This pilot study presents a computational pipeline that bridges anatomical imaging with simulated hemodynamics for personalized BPA planning. The observed trends suggest that lesion morphology may influence the suitability of different virtual planning strategies. This work provides preliminary insights and a methodological foundation for future validation in larger cohorts aimed at enhancing personalized BPA planning for CTEPH.

## Introduction

Chronic thromboembolic pulmonary hypertension (CTEPH), classified as Group IV in the WHO pulmonary hypertension (PH) classification, represents the only curable form of PH through surgical intervention (Kawakami et al., 2016; Matthews and Hemnes, 2016). CTEPH arises as a chronic sequela of pulmonary thromboembolism (PTE), a condition termed post-PTE syndrome (Huisman et al., 2018). The pathophysiology of CTEPH involves a dual mechanism: mechanical obstruction by unresolved, organized thrombi and a secondary, progressive microvasculopathy in unobstructed pulmonary arteries driven by elevated pressure and shear stress (Yandrapalli et al., 2018; M et al., 2017). This leads to a reduction in functional vascular channels, secondary perfusion redistribution, and worsening vascular dysfunction. Clinically, CTEPH manifests as hypoxemia due to ventilation-perfusion mismatch, further complicating patient outcomes (Kapitan et al., 1990).

While pulmonary endarterectomy (PEA) remains the gold standard for eligible patients, its applicability is limited to proximal lesions due to technical challenges and patient-specific contraindications. For patients with distal lesions where surgery is not feasible, balloon pulmonary angioplasty (BPA) has emerged as a minimally invasive alternative, underscoring the need for precise planning in this subgroup (Humbert et al., 2022; Kim et al., 2019; Takano et al., 2024). By disrupting organized thrombi and restoring luminal patency, BPA improves pulmonary hemodynamics and functional capacity (Feinstein et al., 2001; Hoole et al., 2020; Kataoka et al., 2012; Sugimura et al., 2012; Tsugu et al., 2015; Broch et al., 2016). However, BPA outcomes remain variable, influenced by lesion heterogeneity and an incomplete understanding of how localized interventions translate to global hemodynamic improvements (Mahmud et al., 2021).

Current BPA planning relies on anatomical imaging (e.g., CT angiography and ventilation-perfusion scans) and invasive hemodynamic assessments from right heart catheterization (RHC). While these modalities identify stenotic regions, they fail to predict how targeted dilation alters local flow dynamics or which lesions most significantly impact global hemodynamics (Spazzapan et al., 2018; Colebank et al., 2021). For instance, the most common BPA targets—web-like and ring-like stenoses—exhibit distinct mechanical responses to dilation. Web lesions require thrombus compression and neointima formation, whereas ring-like stenoses primarily rely on luminal expansion (Ishiguro et al., 2016; Araszkiewicz et al., 2017; Räber et al., 2019; Shimokawahara et al., 2018; Kopeć et al., 2015; Jin et al., 2020; Kitani et al., 2014; Ogawa et al., 2014). These differences necessitate tailored preoperative strategies to optimize efficacy and minimize complications. Image-based computational fluid dynamics (CFD) offers a powerful tool for predicting hemodynamic changes and elucidating disease mechanisms (Taylor and Figueroa, 2009; Tang et al., 2012; Kheyfets et al., 2013; Kheyfets et al., 2015; Scardulla et al., 2017), yet its application in CTEPH remains limited. Existing studies focus on non-thromboembolic PH (Tang et al., 2012; Kheyfets et al., 2013) or neglect lesion-specific effects (Spazzapan et al., 2018). Recent work by Colebank et al. (Colebank et al., 2021) employed a multiscale model (using one dimension network by reducing the three dimensions vessel geometry to centerlines) to highlight the distinct hemodynamic impact of web-like and ring-like stenoses, emphasizing the need for lesion-specific analysis. Their model suggested that the hemodynamic improvement from a single BPA procedure, particularly in terms of global parameters like pulmonary artery pressure, may be limited, consistent with the clinical observation that benefits often accrue over multiple sessions (M et al., 2017). Consequently, a critical gap persists in translating three dimensions (3D) patient-specific lesion morphology into actionable, spatially-resolved predictions for BPA planning.

To address these challenges, this pilot study introduces and preliminarily assesses two novel virtual BPA (vBPA) approaches: vBPA1, which performs morphology-based restoration of stenotic vessels to approximate normal anatomy, and vBPA2, which simulates rigid homogeneous dilation of target vessels, mimicking idealized balloon expansion. Using patient-specific CT-derived 3D models and CFD simulations, we aim to: 1) quantify hemodynamic changes—including vortex dynamics, wall shear stress, and flow redistribution—induced by virtual BPA; 2) identify factors influencing hemodynamic recovery. We utilize post-BPA imaging and simulations to validate and assess the virtual predictions. This work seeks to explore a computational strategy to bridge the gap between anatomical imaging with functional hemodynamics, offering initial insights toward enhancing BPA planning and advance precision therapy for CTEPH.

## Materials and methods

### Patient cohort and imaging

This retrospective pilot study was designed to develop and preliminarily explore a virtual BPA simulation strategy. We acknowledge that the small cohort size is a limitation for generalizable conclusions but is appropriate for this methodological development stage. The analysis was conducted on three CTEPH patients (presenting with two web-like and one stenotic lesion) and one control subject. Pre-operative clinical imaging (CTPA and ventilation-perfusion scans) identified right-lung lesion dominant in all three CTEPH patients, a frequently observed clinical distribution in CTEPH cohorts, right-sided lesions were selected as the primary targets for the actual BPA procedures. Our virtual planning pipeline was designed to reflect this clinical decision-making process; therefore, the same right-sided lesions were selected as targets for the virtual simulation (vBPA). The control was an individual who underwent computed tomography pulmonary angiography (CTPA) and RHC for suspected pulmonary hypertension, which was subsequently ruled out. This provided a baseline dataset of normal pulmonary arterial geometry and hemodynamics for comparison.

Pre- and post-BPA CTPA scans were acquired using a high-resolution scanner (Siemens SOMATOM Force, Erlangen, Germany) with a 1 mm slice thickness and dual-energy acquisition. Post-BPA follow-up included mid-term RHC assessments (1–4 months). Digital subtraction angiography (DSA) during BPA operation were used for confirmation of ball expansion position. The parameters of subjects are summarized in Table 1. All patient data were anonymized and handled in strict accordance with data protection and confidentiality principles.

**TABLE 1:** Clinical information and RHC parameters.

Variables	Control (Non-CTEPH)	Patient1 (web-lesion)	Patient2 (web-lesion)	Patient3 (stenosis-lesion)
RHC parameters				
mPAP (mmHg)	19	47 vs. 27	60 vs. 39	48 vs. 36
CO (L/min)	5.26	5.6 vs. 2.2	5.4 vs. 6.2	4.3 vs. 4.2
PAWP (mmHg)	13	15 vs. 7	13 vs. 9	12 vs. 11
PVR (dyn·s·cm^−5^)	91	457.14 vs. 727.27	711.11 vs. 361.29	651.16 vs. 514.29
BPA parameters				
Balloon size (Diameter*Length)	/	2.5 mm*30 mm	2 mm*12 mm	2 mm*15 mm; 4 mm*40 mm
Balloon expansion position	/	Right lung lobe: A8-A10	Right lung lobe: A6-A10	Right lung lobe: A4-A7

BPA: balloon pulmonary angioplasty; mPAP: mean pulmonary artery pressure; CO: cardiac output; PVR: pulmonary vascular resistance.

*The parameters of RHC, are presented in the form of a comparison between pre-BPA, and post-BPA (pre-BPA VS., post-BPA).

### Right heart catheterization

For all subjects, RHC and CTPA scans were performed within a 24-h interval. RHC was conducted using a Swan-Ganz catheter (Edwards Lifesciences, Irvine, CA, United States) via jugular vein access. Hemodynamic parameters, including mean pulmonary artery pressure (mPAP), pulmonary arterial wedge pressure (PAWP), cardiac output (CO) measured via thermodilution, and pulmonary vascular resistance (PVR, calculated as (mPAP–PAWP)/CO), were measured pre- and post-BPA. PVR was prioritized as the primary metric for assessing treatment efficacy due to its comprehensive reflection of hemodynamic alterations secondary to vascular remodeling.

### Image segmentation, 3D geometric modeling, and mesh generation

**FIGURE 1:**
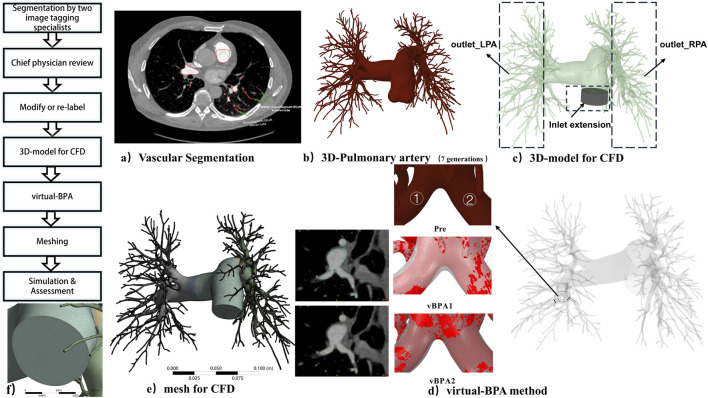
Workflow of segmentation, pulmonary artery model reconstruction, vBPA and comprehensive analysis. **(a)** Vascular segmentation of pulmonary arteries (Patient 1). **(b)** 7^th^-generation pulmonary artery model (Patient 1). **(c)** CFD analysis model, **(d)** Virtual BPA modeling strategies (Patient 1). **(e)** Meshing details (Patient 1). **(f)** A zoomed-in view of the boundary layer mesh.

Vascular segmentation was performed using a semi-automated thresholding workflow on CTPA images to isolate the pulmonary arteries (PA), followed by manual refinement to exclude non-vascular structures (Burrowes et al., 1985). 3D models were reconstructed to include vessels up to the 7th generations (counting the main pulmonary artery as generation 0, the left and right PAs as generation 1, and so forth.) of pulmonary arteries (Kheyfets et al., 2015; Piskin et al., 2020), the labeling of vessels and 3D PA model were verified by a radiologist with over 10 years of experience in cardiothoracic imaging. Figure 1 illustrates the segmentation workflow and the final pulmonary arterial model.

For CFD model preparation, inlet extensions (Capuano et al., 2019) were added to enforce fully developed flow conditions (Kheyfets et al., 2013; Bordones et al., 2018). The morphological characteristics of CTEPH patients were represented as localized stenoses explicitly incorporated into the computational domain. Tetrahedral elements were used for the core volume, with five prismatic boundary layers grown from the vessel walls to resolve near-wall flow gradients (Kheyfets et al., 2015; Kheyfets et al., 2013; Prakash and Ethier, 2001); the growth ratio was set to 1.1. All meshes were generated using ANSYS Meshing (20.0), with the mesh structure displayed in Figure 1e.

A mesh sensitivity study was conducted to ensure the independence of key hemodynamic results from mesh density. Simulations were performed on coarse (∼16 million cells), medium (∼38 million cells), and fine (∼60 million cells) meshes for a representative model. The difference in computed wall shear stress (WSS) over the entire vessel wall and the mean velocity in target regions varied by less than 1% between the medium and fine meshes. Therefore, the medium mesh density was deemed sufficient for all subsequent simulations. A zoomed-in view of the boundary layer mesh at an inlet is provided in Figure 1f.

### Virtual BPA simulation for predictive planning

Two virtual BPA (vBPA) approaches were implemented. vBPA1 (Morphology-Restoring Dilation) aimed to restore stenotic vessels to their normal anatomical shape, approximating the pre-disease state. This was achieved using NURBS (Non-Uniform Rational B-Splines), the centerline and diameter of healthy vessel segments adjacent to a stenosis were extrapolated to reconstruct the pre-disease geometry, guiding a virtual dilation that aimed to restore the lumen to its anatomical norm. This method is conceptually suited for lesions like webs, where the therapeutic goal involves anatomical restoration via thrombus compression and neointima formation. vBPA2 (Rigid Homogeneous Dilation) applied a uniform, rigid dilation along the vessel midline, simulating an idealized balloon expansion without attempting to restore the original vessel morphology. This simpler, computationally efficient approach focuses on the mechanical effect of dilation. For both methods, the inlet flow rate was constrained to match the pre-BPA CO from RHC, and balloon dimensions (e.g., 2.5 mm × 30 mm for Patient 1) were based on clinical records from actual BPA selective according to preoperative anatomical imaging (Figure 1).

### Validation via post-intervention simulation

To isolate the geometric impact of BPA from confounding postoperative CO variability, a vPOST (Virtual Post-BPA) model was created. This model used geometry from post-BPA CT scans but applied the pre-BPA CO as the inlet boundary condition. This standardization allows for an unbiased evaluation of virtual BPA predictions by focusing solely on geometric changes, addressing the interdependence (Qaiser et al., 2023; Kanezawa et al., 2024) of mPAP and CO and the challenge of unknown post-BPA CO in prospective planning. The vPOST simulation thus establishes a benchmark (“ground truth”) for the isolated geometric effect.

### CFD solver settings

The Reynolds-averaged Navier-Stokes (RANS) equations for incompressible flow are considered in all numerical simulations, this approach has been widely applied in computational studies of pulmonary arteries (Tang et al., 2012; Capuano et al., 2019; Whiting and Jansen, 2001) and extensively validated for cardiovascular simulations (Kung et al., 2011; Kung et al., 2014). The continuity (Equation 1) and the momentum (Equation 2) are described as follows:

∇·u=0 (1)

$$\frac{\partial u}{\partial t}+\left(u\cdot \nabla \right)u=-\frac{1}{\rho }\nabla p+\nu \nabla^{2}u$$ (2)

where ρ is the fluid density, u is the velocity vector field, p is the pressure, ν is the kinematic viscosity.

Blood was modeled as a non-Newtonian fluid using the Carreau model (Jafarinia et al., 2022; Pratumwal et al., 2017), described as (Equation 3):

$$\mu =\mu_{\infty }+\left(\mu_{0}-\mu_{\infty }\right){\left[1+{\left(\lambda \dot{\left.\gamma \right)}\right.}^{2}\right]}^{\left(n-1\right)/2}$$ (3)

Where: time is constant *λ* = 3.313 s, zero strain viscosity is *μ*_0_ = 0.056 Pa s, infinite strain viscosity is $\mu_{\infty }$ = 0.0035 Pa s, empirical exponent is *n* = 0.3568.

Boundary conditions included a patient-specific CO (Table 1) at the inlet and 0 Pa pressure at the outlets, a simplifying assumption adopted based on prior studies focused on relative hemodynamic changes in PA (Kheyfets et al., 2013; Kheyfets et al., 2015). Steady-state simulations were used as an initial, computationally efficient approach to analyze the mean hemodynamic state, consistent with the focus on comparing relative changes pre- and post-intervention. The finite volume method (Ansys Fluent 20.0) was employed with a convergence criterion of residuals <10^–6^.

### Hemodynamic analysis

Vortical structures were visualized using the Q-criterion (Hunt et al., 1988) (iso = 0.2, 0.5, 0.8), and colored by normalized helicity density ( $H=\frac{u\cdot \omega }{\left|u\right|\cdot \left|\omega \right|}$ ). WSS analysis utilized the surface area-weighted WSS ( $SAWSS=\frac{1}{A}\int_{A}^{0}WSS\partial A$ ) to mitigate resolution-dependent artifacts, high focal WSS anomalies (>5 Pa) in distal vessels were excluded from SAWSS calculations (Kheyfets et al., 2015). The right-to-left pulmonary artery (RPA/LPA) flow ratios in this study were to assess the pulmonary perfusion imbalances due to pulmonary vascular resistance (Harris et al., 2011).

Velocity profiles at target lesions (labeled as “question1, 2 … n” for different lesions) and downstream regions (labeled as “downstream1, 2 … n”) were analyzed via k-means clustering (n = 4 clusters) to assess hemodynamic recovery, where Clusters 1 and 4 represent near-wall flow and Clusters 2 and 3 represent central flow (ideal parabolic profile). Predictive accuracy was assessed by calculating the Euclidean distance between cluster centroids (pre/vBPA1/vBPA2 vs. vPOST). K-means clustering (n = 4) of 28 vascular regions of three patients generated 336 hemodynamic comparisons (144 lesion-specific), quantified through Euclidean distance analysis. A composite score, integrating weighted Hausdorff (0.2), Wasserstein (0.4), and Procrustes (0.4) residuals for central flow clusters (2/3), was used to evaluate overall method performance. Lower distance or composite score values represent smaller deviation from vPOST benchmark.

## Result

### Vortex dynamics and hemodynamic alterations

**FIGURE 2:**
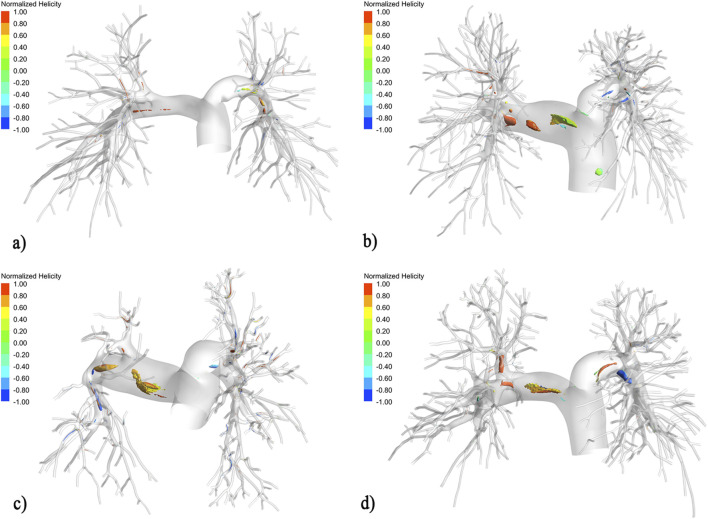
Vortex structures in CTEPH vs. Control, vortex structures visualized by iso-surfaces (iso = 0.5) of Q criterion normalized and colored by normalized helicity. **(a)** Control, mPAP = 19 mmHg. **(b)** Patient 1, mPAP = 47 mmHg. **(c)** Patient 2, mPAP = 60 mmHg. **(d)** Patient 3, mPAP = 48 mmHg.

Consistent with prior observations using 4D flow MRI in PH patients (Reiter et al., 2008), coherent vortical structures were visualized in the main pulmonary artery (MPA) of all three CTEPH patients, whereas the control subject exhibited minimal vorticity (Figure 2). CTEPH patients demonstrated 4–10 times higher vortex volumes across all helicity thresholds (iso = 0.2, 0.5, 0.8) compared to control subject.

Patient-specific analyses revealed distinct patterns. For Patient 1 (Web Lesion), The vPOST model indicated a mild reduction in both the absolute volume and proportional composition of high-helicity vortices post-BPA (volume: 90.31 → 70.88 mm^3^; proportion: 0.99% → 0.76%). Both vBPA approaches accurately predicted this decreasing trend (vBPA1: 88.61 mm^3^, 0.98%; vBPA2: 83.77 mm^3^, 0.93%). For Patient 2 (Web Lesion), in contrast, the vPOST model showed a post-procedural increase in high-helicity vortices (volume: 60.06 → 97.76 mm^3^; proportion: 0.61% → 1.18%). Critically, only the vBPA2 (rigid dilation) method predicted this increase (volume: 66.56 mm^3^; proportion: 0.68%), while vBPA1 predicted a decrease (volume: 55.77 mm^3^; proportion: 0.56%). This vBPA2-predicted augmentation of flow disturbance coincided with the patient’s subsequent hemodynamic rebound, where PVR increased from 361.29 to 571.43 dyn s·cm^-5^ at follow-up. For Patient 3 (Stenosis Lesion), the vPOST model indicated a moderate post-BPA change in high-helicity vortices (volume: 136.20 → 158.26 mm^3^; proportion: 1.68% → 1.89%). Both vBPA methods predicted an increase, with vBPA2 yielding a prediction closer to the vPOST result (vBPA2: 167.95 mm^3^, 2.04%; vPOST: 158.26 mm^3^, 1.89%).

In predicting the critical changes in high-helicity vortex dynamics, vBPA2 demonstrated a trend toward better agreement with the vPOST benchmark across both web and stenosis lesions in our cohort.

### Limited global improvement in flow distribution and wall shear stress

**FIGURE 3:**
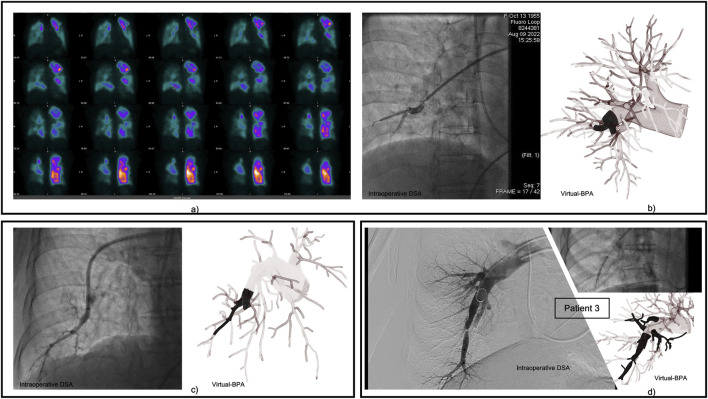
Illustrates the representative vascular locations of three patients during intraoperative DSA for balloon pulmonary angioplasty (BPA). These locations were also the primary focus of virtual BPA in this study. **(a)** preoperative ventilation-perfusion scans of Patient 1, clearly showing that the disease severity in the right lung was higher than that in the left lung. **(b)** Patient 1. **(c)** Patient 2. **(d)** Patient 3.

Pre-BPA RPA/LPA flow ratios (Table 2) were below the control (1.22) in all CTEPH patients (0.75–0.94), reflecting right-lung lesion dominance and consistent with the justifying the prioritization of right-sided interventions based on clinical imaging (Figure 3). The vPOST model showed a post-BPA shift toward a more balanced perfusion in Patients 1 and 3 (ratios: 0.82 and 0.94, respectively), the same shift was also predicted by the vBPA simulations (e.g., Patient 3 vBPA2: 0.88).

**TABLE 2:** Vortex quantification and hemodynamic parameters.

Variables	Control	Patient1				Patient2				Patient3			
		Pre	vBPA1	vBPA2	vPOST	Pre	vBPA1	vBPA2	vPOST	Pre	vBPA1	vBPA2	vPOST
Volume of vortex structure (mm^3^)													
Low	2,068.65	9,093.00	9,020.74	9,005.06	9,348.47	9,840.99	9,939.54	9,726.16	8,298.22	8,099.95	8,325.63	8,219.96	8,371.04
Moderate	320.01	2,239.64	2,324.91	2,307.81	2,542.01	2,298.01	2,342.23	2,368.34	2091.39	2,173.24	2,249.88	2,213.31	2,317.56
High	7.28	90.31	88.61	83.77	70.88	60.06	55.77	66.56	97.76	136.20	155.78	167.95	158.26
Vortex composition (%)													
Low	84.18%	74.38%	73.24%	73.44%	72.05%	76.04%	75.87%	74.97%	73.62%	71.49%	71.11%	71.03%	70.42%
Moderate	15.47%	24.63%	25.77%	25.63%	27.19%	23.35%	23.56%	24.35%	25.20%	26.83%	27.02%	26.93%	27.69%
High	0.35%	0.99%	0.98%	0.93%	0.76%	0.61%	0.56%	0.68%	1.18%	1.68%	1.87%	2.04%	1.89%
Flow rate & SAWSS													
RPA/LPA flow ratio	1.22	0.75	0.75	0.75	0.82	0.84	0.84	0.84	0.83	0.80	0.89	0.88	0.94
RPA_SAWSS (Pa)	1.57	0.29	0.29	0.29	0.31	0.27	0.27	0.27	0.30	0.28	0.29	0.29	0.23
LPA_SAWSS (Pa)	1.38	0.89	0.88	0.88	0.85	0.50	0.50	0.49	0.48	0.46	0.44	0.44	0.39

RPA: right pulmonary artery; LPA: left pulmonary artery; low (helicity) defined as vortex of iso-value = 0.2; moderate (helicity) defined as vortex of iso-value = 0.5; high (helicity) defined as vortex of iso-value = 0.8.

Pre-BPA SAWSS values in both the RPA and LPA of CTEPH patients were lower than in the control subject. Post-BPA SAWSS values remained subnormal (CTEPH range: 0.23–0.88 Pa vs. control: RPA 1.57 Pa, LPA 1.38 Pa).

### Velocity profiles and stenosis impact

**FIGURE 4:**
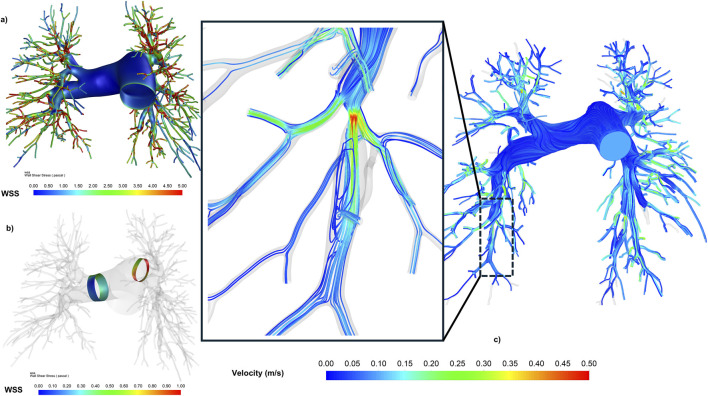
WSS and velocity distribution of pulmonary arteries. **(a)** The global WSS distribution of one representative CTEPH subjects. **(b)** SAWSS (surface area average WSS) over the area by clipping 10-mm circumferential strips of RPA and 5-mm circumferential strips of LPA. **(c)** Velocity streamlines and turbulence downstream of stenosis in a Pre-BPA pulmonary artery.

**FIGURE 5:**
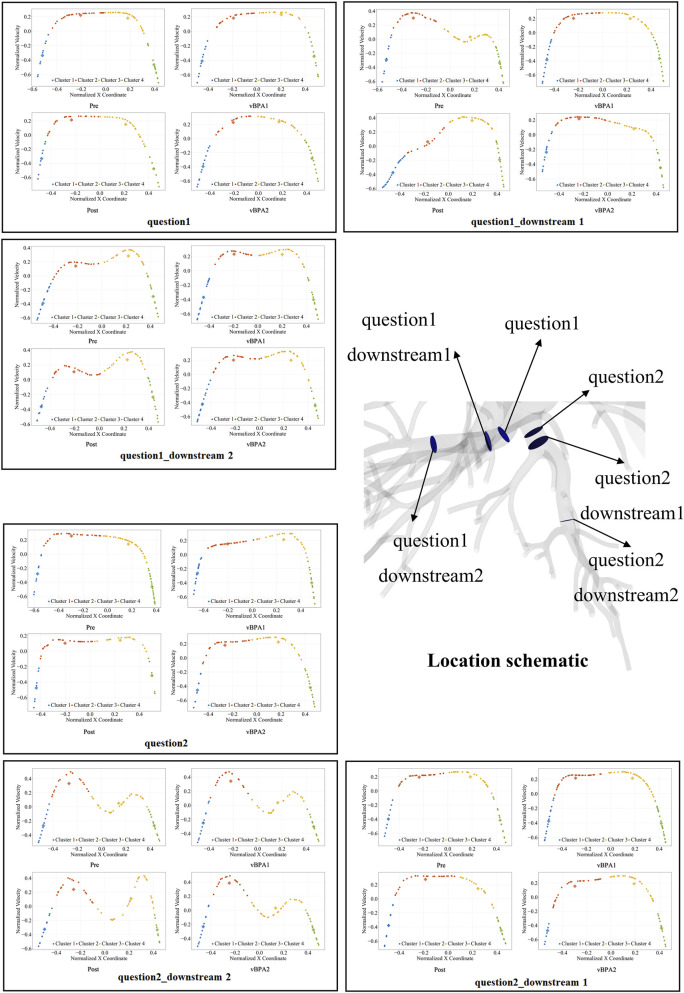
Velocity profiles at target lesions (question) and downstream regions were analyzed via k-means clustering (n = 4 clusters). Cluster 1/4 (blue/green): Near-wall flow development, Cluster 2/3 (orange/yellow): Central flow.

Pre-BPA simulations identified regions of elevated velocity and turbulent flow downstream of stenoses (Figure 4), correlating with clinical intervention targets (Figure 3). Both vBPA models demonstrated restoration of parabolic velocity profiles in target vessels (Figure 5), consistent with normalized flow (Taherian et al., 2017; Hathcock, 2006). However, velocity profiles in distal downstream regions for Patient 1 showed minimal change.

Euclidean distance analysis confirmed localized hemodynamic improvements in the lesion areas for both vBPA methods compared to the pre-operative state (Pre: median 0.794 vs. vBPA1: 0.600 vs. vBPA2: 0.655). However, when incorporating velocity profiles from proximal and distal regions surrounding the lesion, the Euclidean distances for both methods were higher than pre-operative values (Pre: median 0.490 vs. vBPA1: 0.677 vs. vBPA2: 0.596).

### vBPA method performance

**FIGURE 6:**
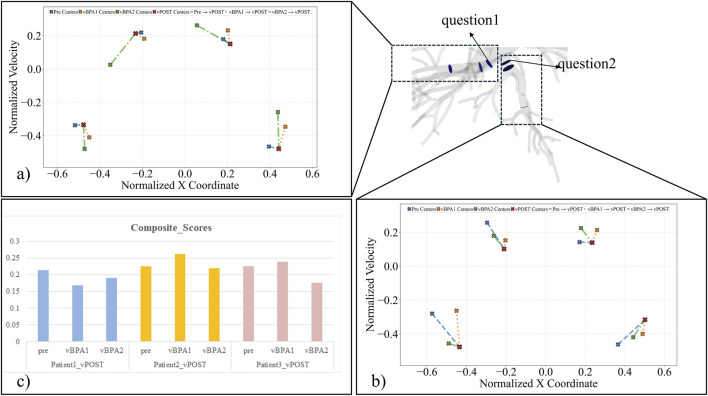
The predictive accuracy of vBPA was assessed by the Euclidean distance and composite score, the distance value represents deviation from vPOST. **(a,b)** The Euclidean distance between the cluster centroids of normalized velocity profiles at different stages (Pre, vBPA1, vBPA2) to the vPOST cluster centroids (n = 4). **(c)** The composite score combined three metrics (Hausdorff distance, Wasserstein distance and Procrustes residual) with weighted factors.

Preliminary results suggested differential performance trends for the two vBPA methods across the analyzed lesion types (Figure 6). For web lesions, vBPA2 (rigid dilation) yielded lower composite scores (0.176–0.220) compared to vBPA1 (0.239–0.262). For the ring-like stenosis lesion, vBPA1 (morphology-restoring) produced a lower composite score than vBPA2 (composite score: 0.168 vs. 0.190). Euclidean distance analysis corroborated these localized improvements while highlighting compensatory flow fluctuations in distal regions.

## Discussion

This pilot study presents an initial exploration of two patient-specific virtual BPA strategies, using post-intervention imaging to assess their simulated hemodynamic predictions. Our primary aim was to illustrate the potential of such a computational approach and share preliminary observations that may inform future research.

The observed vortex dynamics in our CTEPH patients align with prior 4D flow MRI studies, such as that by Reiter et al. (Reiter et al., 2008), which described coherent vortices in the MPA of PH patients. This may consistency supports the physiological relevance of our CFD models in capturing key flow disturbances associated with elevated pulmonary pressures, since we also found the high-helicity vortex composition of the three CTEPH higher than the control in all states (Pre,vBPA, vPOST). The evolution of high-helicity vortices may offer predictive insight to illustrate BPA’s primary mechanism appears, the most salient observation was in Patient 2, where the vBPA2 simulation uniquely predicted a post-procedural increase in high-helicity vortices. This prediction aligned with the patient’s subsequent clinical course of PVR rebound after initial improvement. High-helical flow (regarded as a disturbed flow) is associated with increased endothelial cell activation and pathological shear stress patterns (Cybulsky and Marsden, 2014). Its sustained presence, therefore, could be a hemodynamic biomarker for incomplete vascular recovery or ongoing microvasculopathy, aspects not directly measured by standard RHC. This case suggests a potential temporal decoupling between acute afterload reduction (captured by PVR) and the persistence of abnormal local flow patterns that may drive later vascular remodeling—an insight that merits further investigation in larger studies.

The performance trends of the two vBPA methods, while preliminary due to the small sample, offer an intriguing conceptual insight. The trend suggesting vBPA2 (rigid dilation) for web lesions may reflect its simulation of the immediate, mechanical stretching of organized thrombi—a primary mechanism of BPA in such lesions (Shimokawahara et al., 2018). Conversely, the trend for vBPA1 in the ring-like stenosis aligns with a goal of anatomical reconstruction. vBPA1 (morphology-restoring) for the ring-like stenosis aligns with the goal of anatomical lumen reconstruction. This dichotomy hints that the underlying mechanical action of BPA—compression versus expansion—might benefit from distinct modeling philosophies, a hypothesis that could guide the development of more sophisticated virtual planning tools.

Our findings regarding WSS are consistent with established literature reporting WSS attenuation in pulmonary hypertension (Kheyfets et al., 2015; Spazzapan et al., 2018; Bordones et al., 2018; Piskin et al., 2020). The substantially lower SAWSS values in CTEPH patients pre-BPA, which remained subnormal post-BPA, reflect the pervasive vascular dysfunction. The modest hemodynamic improvements predicted by a single virtual intervention are consistent with computational work by Colebank et al. (Colebank et al., 2021) that a single, localized BPA procedure, while improving conduit patency, cannot normalize the systemic (global) environment, explaining why clinical benefits typically accrue over multiple sessions (Hug et al., 2021).

The increased Euclidean distance in peri-lesional regions (downstream of lesion) post-simulation may reflect both the localized nature of the intervention and the current models’ limitations in capturing systemic compensatory mechanisms.

## Limitation and future directions

Our study has several limitations that frame the interpretation of these pilot results. First, the small cohort size of three patients precludes definitive conclusions about lesion-specific strategies and generalizability. Second, the use of steady-state simulations and simplified outflow boundary conditions (0 Pa), while common for comparative studies (Kheyfets et al., 2015), neglects pulsatile effects and wave propagation. Third, the current models do not incorporate vascular wall elasticity, active remodeling, or changes in distal vascular resistance, which are critical factors in the dynamic response to BPA, as hinted by the CO drop in Patient 1 and the PVR rebound in Patient 2. The vPOST model, while a useful benchmark, also assumes no change in cardiac output post-BPA, which is a clinical variable. Despite these limitations, this work establishes a crucial methodological foundation. Future research should focus on validating these findings in larger, prospective cohorts. Incorporating unsteady flow simulations, coupled multiscale boundary conditions, and potentially fluid-structure interaction would enhance physiological fidelity. Most importantly, a prospective study where vBPA predictions are made pre-operatively and compared to both post-BPA imaging and long-term clinical outcomes is the essential next step to evaluate clinical utility. Furthermore, exploring the integration of this hemodynamic data with other modalities, such as intravascular imaging or perfusion scans, could create a more comprehensive planning tool.

## Conclusion

This pilot study introduces a computational pipeline for simulating BPA in CTEPH and shares preliminary observations from its application in a small case series. The results suggest the potential of such simulations to reveal complex hemodynamic responses and hint that lesion morphology might influence the choice of virtual planning strategy. By providing these initial insights, this work aims to contribute to the ongoing development of more personalized and predictive planning tools for BPA, the clinical utility of which must be established through future validation in larger cohorts.

## Institutional Review Board (IRB) approval

This study was conducted under a protocol approved by the Institutional Review Board of Sir Run Run Shaw Hospital, Zhejiang University School of Medicine, Hangzhou, China (Number: 2022-0083).

## Data Availability

The original contributions presented in the study are included in the article/supplementary material, further inquiries can be directed to the corresponding authors.

## Glossary

BPA: Balloon Pulmonary Angioplasty
CFD: Computational Fluid Dynamics
CO: cardiac output
CT: Computed Tomography
CTEPH: Chronic Thromboembolic Pulmonary Hypertension
CTPA: Computed Tomography Pulmonary Angiography
DSA: Digital Subtraction Angiography
LPA: left pulmonary artery
mPAP: mean pulmonary artery pressure
MPA: main pulmonary artery
NURBS: Non-Uniform Rational B-Splines
PA: pulmonary arteries
PAWP: pulmonary arterial wedge pressure
PEA: Pulmonary Endarterectomy
PH: Pulmonary Hypertension
PTE: pulmonary thromboembolism
PVR: pulmonary vascular resistance
RANS: Reynolds-averaged Navier-Stokes
RHC: Right Heart Catheterization
RPA: right pulmonary artery
SAWSS: surface area-weighted wall shear stress
vBPA: Virtual Balloon Pulmonary Angioplasty
vPOST: Virtual Post-BPA
V/Q: ventilation-perfusion scans
WSS: wall shear stress
